# Large-scale patterns of number use in spoken and written English

**DOI:** 10.1515/cllt-2022-0082

**Published:** 2023-03-27

**Authors:** Greg Woodin, Bodo Winter, Jeannette Littlemore, Marcus Perlman, Jack Grieve

**Affiliations:** Department of English Language and Linguistics, University of Birmingham, Birmingham, United Kingdom

**Keywords:** big data, number frequencies, numerical cognition, register studies, rounding

## Abstract

This paper describes patterns of number use in spoken and written English and the main factors that contribute to these patterns. We analysed more than 1.7 million occurrences of numbers between 0 and a billion in the British National Corpus, including conversational speech, presentational speech (e.g., lectures, interviews), imaginative writing (e.g., fiction), and informative writing (e.g., academic books). We find that four main factors affect number frequency: (1) Magnitude – smaller numbers are more frequent than larger numbers; (2) Roundness – round numbers are more frequent than unround numbers of a comparable magnitude, and some round numbers are more frequent than others; (3) Cultural salience – culturally salient numbers (e.g., recent years) are more frequent than non-salient numbers; and (4) Register – more informational texts contain more numbers (in writing), types of numbers, decimals, and larger numbers than less informational texts. In writing, we find that the numbers 1–9 are mostly represented by number words (e.g., ‘three’), 10–999,999 are mostly represented by numerals (e.g., ‘14’), and 1 million–1 billion are mostly represented by a mix of numerals and number words (e.g., ‘8 million’). Altogether, this study builds a detailed profile of number use in spoken and written English.

## 1 Introduction

People use some numbers more often than others. For example, English speakers tend to use numbers like 3, 100, and 2022 more often than numbers like 43, 104, and 3022. More generally, smaller numbers may be used more often than larger numbers, round numbers (e.g., multiples of ten) more often than unround numbers, and culturally salient numbers (e.g., recent years) more often than non-salient numbers. People may also use numbers more often when communicating precise information, or in situations where presenting facts in an informative manner is important. Exploring the frequency with which different numbers are used can reveal the numbers people deem important to communicate about, in what contexts, and why. The present study investigates patterns of number use in spoken and written English and explores the primary factors that contribute to these patterns. Using the 100 million word British National Corpus (BNC Consortium 2007), we identified over 1.7 million occurrences of numbers from 0 to a billion and analysed their frequency in relation to four factors: magnitude, roundness, cultural salience, and register. In written texts, we explored the format in which numbers of different magnitudes are expressed, i.e., as numerals (e.g., ‘140’), number words (e.g., ‘three’), or a mix of both (e.g., ‘1 million’). Overall, this paper reports the largest, most comprehensive corpus analysis of number use to date.

## 2 Background

Several factors are believed to influence the frequency with which people use different numbers. One is *magnitude*: studies have shown that people use smaller numbers more frequently than larger numbers (Coupland 2011; Dehaene and Mehler 1992; Dorogovtsev et al. 2005; Jansen and Pollmann 2001). A possible explanation for why people discuss smaller numbers more frequently is that smaller quantities are encountered more often and are more easily countable, making them more relevant to discuss (Cummins 2015: 32). For example, we are more likely to encounter a group of three people than a group of 27, and even if we do encounter the larger group, the exact number may be unknown, or irrelevant to the point that a simple non-numerical description such as “a large group” or “several people” may suffice. The bias toward using small numbers may create a feedback loop in which smaller numbers are encountered more often and learnt earlier, which may cause them to be used more frequently still (Rath 1966). Dehaene and Mehler (1992) also consider the possibility that people discuss small numbers more often because these numbers are easier to mentally process, citing magnitude comparison tasks in which smaller numbers are identified more quickly than larger ones (Buckley and Gillman 1974; Dehaene 1989). Another aspect to consider is that we conventionally use scales to keep numbers small (Coupland 2011: 34–35) – for example, we can avoid having to talk about 600 seconds by using a larger temporal unit, like 10 minutes.

The second factor that influences number frequency is *roundness:* research has shown that round numbers are used more frequently than unround numbers of a similar magnitude (Coupland 2011; Dehaene and Mehler 1992; Dorogovtsev et al. 2005; Jansen and Pollmann 2001). In decimal number systems – those based on the number ten (including tenths and powers of ten), such as the English system – round numbers are typically considered to be multiples of ten and sometimes five (e.g., Dorogovtsev et al. 2005; Sigurd 1988). One possible reason why round numbers are used more frequently is that they can be used approximately in a practice known as *rounding*, where the nearest round number is used in place of the real value (Cummins 2015: 20; Jansen and Pollmann 2001; Krifka 2009; Sigurd 1988). For example, instead of saying that a lecture has 99 attendees, a speaker may round 99 up to 100. In instances like this, round numbers can represent a range of values – they have a larger *pragmatic halo* (see Lasersohn 1999) than unround numbers, which usually only represent a single precise value. For example, it is generally acceptable to use ‘100’ to denote the number 103, while it is generally unacceptable to use ‘103’ to denote the number 100. In other cases, the use of rounding may be explicitly signaled, such as if a speaker were to say that a distance is “a hundred and twenty miles, to the nearest ten miles” (example from the British National Corpus: BNC Consortium 2007). The increased flexibility in the use of round numbers may help explain why they have been found to be used more frequently than unround numbers. Additionally, the nearest round number can be used as a benchmark indicating some level of completion (Dehaene 1997, Ch. 4; Gunasti 2016). For instance, if there are 99 people at a lecture, someone may write that there are “nearly 100 people”, whereas if there are 103 people, they may instead write that there are “over 100 people”.

The reason that multiples of ten and five are treated as round numbers may be that they are psychologically salient (Van der Henst and Sperber 2004), making these numbers more cognitively accessible, simpler, and less cognitively costly compared to other numbers (Dehaene 1997; Krifka 2009; see Cummins 2015: 32). The psychological salience of these numbers may be due to their structural prominence within the decimal system, which may have deeper roots in finger counting practices (Butterworth 1999; Dehaene 1997; see Cummins 2015: 33): the number of fingers on each hand may be the basis of decimal systems (e.g., Bender and Beller 2012; Ifrah 1987; Wiese 2003). Due to this special status, cultural artifacts and customs are often based on these values. For example, packs of ten items are more common than packs of 11 items (Cummins 2015: 36), and we celebrate jubilees on the 5th, 10th, 25th, and 50th (etc.) anniversaries, rather than the 6th, 12th, 18th, and so on (Jansen and Pollmann 2001). These cultural artifacts and customs may create a feedback loop in which multiples of ten and five are encountered even more often (Chrisomalis 2020: 35), further enhancing the psychological salience of these numbers (Cummins 2015: 36).1The idea that roundness is determined by the base of the mathematical system means that the numbers considered round differ between different systems. We can see evidence of this in sexagesimal systems (base 60) used, for example, in the measurement of time. In this time-telling context, 15 and 30 may be round numbers because they are, respectively, a quarter and half of 60 s or min (Dehaene and Mehler 1992; Jansen and Pollmann 2001). Similarly, 90, 180, and 360 may be round numbers in the measurement of angles. However, in this paper we focus on round numbers in decimal systems, the predominant system in Western cultures.


People use round numbers in an approximate way for several different reasons. First, the precise number is often deemed unnecessary to mention. For example, while it might be helpful to have a ballpark figure of the number of people that will be attending your lecture, the precise number may be practically irrelevant. By using round numbers in such contexts, language users conform to Grice’s (1975) maxims, being only as precise as is necessary to achieve the aims of the interaction (Gibbs Jr. and Bryant 2008; Van der Henst et al. 2002). Unnecessary precision may also be seen as pedantic (Beltrama et al. 2022; see also Cotterill 2007; Lin 2013; McCarthy 2006: 22), exerting additional social pressure toward rounding in certain contexts. Second, people often use round numbers imprecisely when the exact number is unknown, such as when estimating the size of a crowd (e.g., ‘a thousand people’), using a round number to reduce commitment to a precise value (Ruud et al. 2014). This strategy works because people usually interpret round numbers approximately, unless they are modified by words such as ‘exactly’ (Krifka 2009; Lasersohn 1999). Finally, another reason that can motivate the use of round numbers is strategic manipulation, such as when stating that a university is a ‘top ten university’ (a round number) as opposed to a ‘top six university’ (an unround number in context), because the latter expression yields the disadvantageous implicature that the university is exactly 6th place (Cummins and Franke 2021).

Corpus studies have shed light on the two roles of magnitude and roundness in numerical communication. Dehaene and Mehler (1992) investigated number words (e.g., ‘three’, ‘sixteen’, ‘eighty’, ‘thousand’) from 0 to a billion in a corpus of over 1 million American English words (Francis and Kučera 1982). The results showed that number frequencies decline with numerical magnitude, but that round numbers 10, 20, 50, and 100 are used more frequently than other numbers of a similar magnitude. Jansen and Pollmann (2001) reported similar results for numerals and number words in the range 2–1,000 in a 40 million word corpus of 1994 volumes of English newspaper *The Times.* Coupland (2011) uncovered a similar pattern for number words from 1 to 20 in the 100 million word British National Corpus (BNC Consortium 2007), and for both numerals and number words from 1 to 100 on the internet. Lastly, though not framed explicitly as a corpus study, Dorogovtsev et al. (2005) found that frequencies of web pages containing positive numerals declined with numerical magnitude, but that frequencies were relatively higher for pages containing powers of ten (i.e., round numbers in the decimal system).

Some authors have argued that roundness is a matter of degree, rather than being absolute. For example, Sigurd (1988) proposed a model of roundness where powers of ten (e.g., 10, 100, 1,000) and halves (e.g., 5, 50, 500) and quarters (e.g., 2.5, 25, 250) of these powers are ‘rounder’ than other numbers. Jansen and Pollmann (2001) developed Sigurd’s model, showing empirically that numbers are used more frequently if they have one of the properties of 10-ness (10, 20, 30, … 100, 200, 300, …), 2-ness (20, 40, 60, … 200, 400, 600, …), 2.5-ness (25, 50, 75, … 250, 500, 750, …), and 5-ness (50, 100, 150, … 500, 1,000, …). Notably, 10-ness, 2-ness, 2.5-ness, and 5-ness do not simply refer to numbers that are divisible by these respective factors; rather, they refer to numbers that equal an integer no greater than 9 when divided by 1, 2, 2.5, or 5 multiplied by a power of 10. For example, 200 has 10-ness because it equals 2 when divided by 100 (1 × 10^2^), and 750 has 2.5-ness because it equals 3 when divided by 250 (2.5 × 10^2^). Jansen and Pollmann (2001) argue that these numbers are used more often due to humans having a “natural propensity” (p. 201) for doubling and halving the base of the mathematical system (e.g., powers of ten in a decimal system), and, in the case of 2.5-ness, halving again.

Two questions regarding roundness remain unanswered. First, it is not clear if multiples of ten and five *without* 10-ness, 2-ness, 2.5-ness, or 5-ness (e.g., 1,010, 70,515, 944,500) are used more often than non-multiples, or if it is only those multiples of ten and five with 10-ness, 2-ness, 2.5-ness, or 5-ness (e.g., 70, 250, 600) that are used more often. Second, Jansen and Pollmann (2001) report that numbers with more roundness properties (e.g., 40 has 10-ness, 2-ness, and 5-ness; 300 has 10-ness and 5-ness) are used more frequently than numbers with fewer. However, as Cummins (2015: 34) points out, we do not know whether certain of these properties are more important predictors of number frequency than others. For instance, 10-ness may be a more important predictor of number frequency than 2-ness, or vice versa. Table 1 shows examples of numbers with each of the roundness properties discussed in this paper.

**Table 1:** Examples of numbers with properties associated with being a round number: Multiple of 5, Multiple of 10, 10-ness, 2-ness, 2.5-ness, and 5-ness. Numbers can have more than one of these properties at once (e.g., 100 has all six roundness properties; 60 is a multiple of five and ten and has 10-ness and 2-ness).

Roundness property	Examples	
	Has property	Does not have property
Multiple of 5	15	57
	765	313
	94,300	61,692
	525,635	865,387

Multiple of 10	90	96
	760	592
	73,270	92,133
	198,250	285,989

10-ness	80	47
	300	523
	3,000	18,341
	600,000	527,374

2-ness	60	64
	200	897
	1,400	31,128
	100,000	587,084

2.5-ness	50	58
	125	903
	10,000	87,882
	500,000	140,770

5-ness	100	84
	4,500	751
	45,000	17,329
	350,000	187,772

The two factors of magnitude and roundness may interact: people may round larger numbers to a greater extent than smaller numbers (see Coupland 2011). If so, this aspect of numerical communication may reflect numerical cognition, which becomes less precise for larger quantities. We can precisely quantify sets of three or four objects or fewer, which is known as *subitization* (Butterworth et al. 2008; Gordon 2004; Pica et al. 2004). However, above this limit, this quantification ability is approximate; we cannot, for example, quantify a crowd at a glance (Cheyette and Piantadosi 2019; McCrink and Wynn 2007; Xu and Spelke 2000). This quantification ability is increasingly imprecise for larger sets, which is described by the Weber-Fechner law: the just-noticeable difference between quantities is linearly related to their ratio. According to this law, sets of 110 and 120 (ratio = 11:12) are harder to discriminate than sets of 10 and 20 (ratio = 1:2), despite each pair differing by an equal number (10) (DeWind et al. 2015; Shepard et al. 1975). If we find it difficult to discriminate larger sets, we may be unlikely to communicate the differences between these sets precisely, leading to a more approximate use of round numbers at higher magnitudes.

In addition to magnitude and roundness, a third factor that influences the frequency with which numbers are used is *cultural salience*. For example, studies have reported that recent years (e.g., 2021) are discussed more often than less recent years (e.g., 1788), probably because more recent years tend to be more relevant to present discussion (Pollmann 1998; Pollmann and Baayen 2001). It is also possible that numbers referring to significant dates and periods in human history, such as 1066 (the Battle of Hastings), 2019 and 2020 (the beginning of the Covid-19 pandemic), and 1939–1945 (World War II) are used more frequently today than other numbers. Moreover, Coupland (2011) finds that numbers with repeated numerals, like 99, 11, and 44, are often used in product names, like the Xbox 360 game ‘Ninety-Nine Nights’, perhaps for their phonological alliteration, aesthetic appeal, and ‘coolness’. Other numbers are imbued with numerological significance and so may be expected to be used more or less often on this basis. For example, in Christian cultures, the number seven is associated with perfection and the Christian God (and is also the number of days in a week), while 666 is associated with the Christian devil (Ayonrinde et al. 2021). Dehaene and Mehler (1992) also report that the number 13 is used less frequently than 12 or 14, perhaps because 13 is deemed unlucky in Western cultures, to the extent that floor numbering systems in many buildings skip the number 13 (Pokryshevskaya and Antipov 2015).

Finally, a fourth factor that may affect number frequencies is *register.* Registers are varieties of language use that are linked to a communicative context or goal (e.g., Trudgill 1983: 101). Whereas the other three factors influence the frequency of certain numbers, this factor relates to how number use differs in different registers. One dimension of register variation is modality – speech versus writing – and studies have revealed differences in language use across more specific spoken and written registers, such as conversational speech (Biber 2009b; Biber et al. 1999, Ch. 13; Conrad and Biber 2009) and informational writing (Biber 2009a, 2009b; Biber et al. 1999; Biber and Clark 2002; Biber and Gray 2010; Conrad and Biber 2009). Neumann (2014) also reports on linguistic differences between instruction manuals, novels, letters to shareholders, and other registers, while Egbert and Mahlberg (2020) show differences between narration and speech in novels, and Love et al. (2019) find differences in language use (e.g., turn length, word length, modal verbs) across different activity types and locations (e.g., a family playing a board game at home vs. colleagues discussing a project at work). There is reason to believe that number use may also vary across registers according to whether the primary communicative goal is to inform. For example, Zillman and Brosius (2000: 42) report that 44% of the news articles they investigated included numbers, like percentages, amounts, and proportions. Koetsenruijter (2011) finds that the use of numbers in news texts enhances the perceived credibility of the information being communicated. Porter (1995) also argues that statistics are used in academic and professional practice due to their perceived objectivity (see Barchas-Lichtenstein et al. 2022 for a similar argument pertaining to journalism). Moreover, quantification may be necessary to convey precise numerical information, for example, in factual reports or financial transactions (Coupland 2011). For these reasons, there may be more of an emphasis on numbers in more informational registers, such as news broadcasts, academic writing, and journalism, compared with less informational registers, like casual conversation and fiction writing.

Investigating number use in written registers raises the additional question of how these numbers are expressed – as numerals (e.g., ‘1,000,000’), number words (e.g., ‘one million’), or a mix of both (e.g., ‘1 million’). Throughout history, there have always been multiple ways of writing a number in all literary traditions (Chrisomalis 2020, Ch. 6). Many style guides, like the Office for National Statistics’ (2022), prescribe number words for numbers 0–9, numerals for numbers 10–999,999, and a mix of numerals and number words for numbers above this range. We might wonder, then, whether the representational formats chosen by authors reflect these style guides, and hence whether numbers of different magnitudes tend to be represented in different formats. On the internet, Coupland (2011) finds that numerals outnumber number words by a ratio of about 4:1, and that this ratio increases rapidly to approximately 200:1 by the number 99. However, the number words explored in this study were exclusive to English, while the numerals 1, 2, 3, and so on are used in many different languages (Chrisomalis 2020). Thus, we may observe a different pattern with proportionally more reliance on number words in a monolingual corpus.

In this study, we examine the frequency with which different numbers are used in a large corpus of spoken and written English. We observe how these frequencies are affected by magnitude, roundness, cultural salience, and register, and within written registers, we investigate how different formats (numerals, number words, mixed numbers) are used to represent numbers of different magnitudes. Our study, based on over 1.7 million numbers from 0 to a billion used in English in the 100 million word British National Corpus (BNC Consortium 2007), makes the following contributions. First, it replicates previous corpus studies of number use in regard to magnitude and rounding (Coupland 2011; Dehaene and Mehler 1992; Dorogovtsev et al. 2005; Jansen and Pollmann 2001). Second, it extends work by Sigurd (1988) and Jansen and Pollmann (2001) by presenting an updated model of round numbers, exploring which roundness properties matter more than others in determining a number’s frequency, controlling for its magnitude. This insight suggests that round numbers are not created equal – some are ‘rounder’ than others. Third, it investigates cultural salience as a general factor that may influence number frequencies, extending previous results pertaining to recent years (Pollmann 1998; Pollmann and Baayen 2001), and numbers with numerological significance (e.g., 13) (Dehaene and Mehler 1992) or aesthetic appeal (e.g., 99) (Coupland 2011). Fourth, it introduces register as a novel factor that influences number frequencies, investigating whether number use differs across more informational and less informational texts. Finally, it shows that, in writing, numbers of different magnitudes tend to be represented in different formats (numerals, number words, mixed numbers). In doing so, this study builds a detailed profile of number use in spoken and written English.

## 3 Methodology

### 3.1 The corpus

The British National Corpus (BNC; BNC Consortium 2007), collated from 1991 to 1994, is a 100 million word corpus of spoken and written British English from the late 20th century. The corpus was slightly revised prior to the 2001 and 2007 releases (hence the citation date above) but no new texts were added. The corpus includes samples of 45,000 words from longer texts, whereas texts under the 45,000 word limit are included in full. Samples were taken to avoid overrepresenting idiosyncratic texts and obtain a wide cross-section of British English. The corpus contains both single-author texts (e.g., monographs) and multi-author texts (e.g., magazine articles).

The spoken (10%) and written (90%) subcorpora are divided into four categories, including two spoken categories (conversational speech and presentational speech) and two written categories (imaginative writing and informative writing). The conversational speech subcorpus was collected via demographic sampling, aiming to capture a representative spread of language users according to age, gender, social group, and region. In contrast, what we call presentational speech – ‘context-governed speech’ according to the BNC nomenclature – encompasses presentations to an audience, such as broadcast interviews, lectures, and legal proceedings. Regarding the written subcorpora, imaginative writing includes literary and creative fictional works, while informative writing includes non-fiction works in domains like applied science, finance, and world affairs. Broadly, presentational speech and informative writing are focused on communicating information, whereas conversational speech and imaginative writing are less informational. For more information about the BNC, see http://www.natcorp.ox.ac.uk/corpus/index.xml.

### 3.2 Software

The programming language Python (version 3.7) (Python Software Foundation 2021) was used inside the integrated development environment PyCharm (version 2021.1.1) (JetBrains 2021) to extract numbers from the British National Corpus (BNC Consortium 2007). The following built-in Python libraries were used: Re (version 2.2.1), Time, OS, and IterTools (all: Van Rossum 2020). The following external Python libraries were used: NumPy (version 1.20.2) (Harris et al. 2020), Pandas (version 1.2.4) (McKinney 2010), NLTK (version 3.6.1) (Bird et al. 2009), BeautifulSoup (version 4.9.3) (Richardson 2007), Requests (version 2.25.1) (Chandra and Varanasi 2015), Word2Number (Batorsky et al. 2021), and Num2Words (Dupras et al. 2021).

The statistical programming language R (version 4.0.3) (R Core Team 2020) was used inside integrated development environment RStudio (version 2022.7.1.554) (RStudio Team 2022) to assist with the number identification and perform the main statistical analyses. The following R packages were used: tidyverse (version 1.3.0) (Wickham et al. 2019), brms (Bürkner 2017, 2018), ggpubr (version 0.4.0) (Kassambara 2020), scales (Wickham and Seidel 2020), car (version 3.0.11) (Fox and Weisberg 2019), ggmcmc (version 1.5.1.1) (Fernández-i-Marín 2016), tidybayes (version 3.02) (Kay 2022), and ggrepel (version 0.9.1) (Slowikowski 2021). All data, analysis scripts, and information about the procedure detailed in this section are stored in an OSF repository (https://osf.io/ze9vk/).

### 3.3 Number identification and data processing

We identified integers and decimals ≥0 (i.e., no negative numbers) in the 4,054 texts that comprise the BNC. Unlike previous corpus studies (Coupland 2011; Dehaene and Mehler 1992; Dorogovtsev et al. 2005; Jansen and Pollmann 2001), which searched for specific numbers in their respective corpora, we used natural language processing to parse the BNC texts and capture all words identified as numbers. As a result, we identify a greater range of numerical language than the aforementioned studies, but we have to perform extra steps to process the data, which we detail in this section. All decisions made in regard to number identification and data processing are fully documented with reproducible scripts at the OSF repository associated with this paper (https://osf.io/ze9vk/).

To understand our data processing decisions, we must differentiate between three senses in which numbers are used: cardinal, ordinal, and nominal (Wiese 2003; Nieder 2005). Cardinal numbers refer to quantity or numerosity, such as the number of apples in a basket. Ordinal numbers refer to numerical rank in an ordered list, such as the top ten universities in the world. Finally, nominal numbers are used as names or identifiers, as in telephone numbers or bus numbers, with no quantity or order necessarily being implied. Cardinal numbers are arguably the most prototypical number sense, and are what we focus on in this paper, motivating our analysis of numerical magnitude and roundness. For this reason, we do not analyse numbers that are explicitly marked as being ordinal (e.g., ‘sixth’, ‘6th’), in line with previous studies (Coupland 2011; Jansen and Pollmann 2001; but see Dehaene and Mehler 1992). Thus, we focus here on a *specific form* of numbers: the most unmarked forms of number words (‘one’, ‘two’, ‘three thousand’, etc.) and numerals (‘1’, ‘2’, ‘3,000’, etc.). We also exclude some instances of nominal numbers, as discussed below. However, it is impossible to make sure we only identify cardinal numbers in such a large dataset, as doing so would require checking each number in its context of use. The concern that our dataset is ‘contaminated’ by non-cardinal numbers is, however, shared with all previous analyses of number word and numeral frequencies (Coupland 2011; Dehaene and Mehler 1992; Dorogovtsev et al. 2005; Jansen and Pollmann 2001). A benefit of our study is that our analysis explicitly acknowledges non-cardinal uses of numbers by investigating numbers whose frequency cannot be predicted accurately by focussing solely on magnitude and roundness (e.g., some culturally salient numbers).

We used the NLTK Python library to tag words in each text for whether they were numbers. The NLTK library tags each word individually, meaning that numbers comprising multiple words (e.g., ‘twenty five’ = two words) were initially tagged as separate numbers (e.g., ‘twenty’ and ‘five’). To capture the full number, we grouped together words tagged as numbers that were adjacent in the same text. We included the words ‘and’ and ‘point’ in this grouping to capture number words containing a conjunction (e.g., ‘one hundred and one’) or decimal values (e.g., ‘two point one’). Hyphenated number words, like ‘twenty-five’, were captured as a whole. NLTK was able to differentiate pronominal uses of number word ‘one’ (e.g., ‘as one does’) from numerical uses (e.g., ‘one person’), which is a novel aspect of our methodology: these uses of ‘one’ are confounded in Dehaene and Mehler’s (1992) and Coupland’s (2011) corpus analyses of number words, and Jansen and Pollmann (2001) chose not to look at the number word ‘one’ for this reason.

This number identification procedure identified 1,918,146 items (called as such because some items were not numbers, or included more than one number; see below). We then removed items that did not meet our definition of non-negative numbers that were not explicitly marked as ordinals. Also removed were items that were incorrectly tagged as numbers, did not refer to a specific number, or were not relevant for other reasons. In particular, we removed 146,797 items containing a mixture of both numerals and letters or other characters, including the aforementioned ordinals (e.g., ‘6th’), year ranges (‘1990s’), file names (e.g., ‘011207.tmp’), digital times (e.g., ‘06:00’), and ratios (e.g., ‘2:1’). Furthermore, we removed number words like ‘twelve fifty’ (6,016 items) that were ambiguous between different types of numbers, such as prices (e.g., £12.50), times (e.g., ‘12:50’) and years (e.g., ‘1250’); non-numerical words that were erroneously tagged as numbers (e.g., ‘year’, ‘ze’: 3,855 items); and vague numbers that did not refer to a precise value (e.g., ‘twenty odd’: 9 items). Finally, we excluded 5 items that included numerals preceded or followed by a full stop and then followed by a multiplier word (e.g., ‘1 million’), and 9,407 items beginning with ‘0’ that resembled binary codes (e.g., ‘00010’), digital times (e.g., 07.15), or telephone numbers (e.g., 0207521133).2We have no systematic way of identifying binary codes, digital times, and telephone numbers that begin with a number other than 0, as these look the same as other numbers (e.g., 1001, 20.55, 3669734), so there may be some of these numbers in the final dataset. Following these exclusions, 1,750,086 items remained.

For the purposes of the data analysis, the number words we identified had to be translated into numerals so they could be recognised by R as numbers rather than as character strings. The Word2Num library in Python was used to translate the number words we identified into numerals. To verify each translation, we used the Num2Words library to back-translate the numerals into number words. We deemed the translation accurate if the back-translated number word matched the original number word. We manually translated some numbers that Word2Num was not able to automatically translate, including mixed numbers comprising a numeral followed by a multiplier word (e.g., ‘1.5 million’ = 1,500,000) and numbers written in a different format (e.g., ‘seventy one hundred’ rather than ‘seven thousand one hundred’ = 7,100). Furthermore, number words with an empty decimal place (e.g., ‘one point zero’) were manually translated into integers (e.g., ‘1’).

Even after these manual translations, we were unable to translate some items into numerals. Consulting these untranslatable items, we saw that many were not individual numbers, but were concatenations of multiple numbers (e.g., ‘one hundred one thousand’ = ‘one hundred’ and ‘one thousand’). These multi-number items were a by-product of our grouping together words tagged as numbers that were adjacent in the same text. To capture the individual numbers in these multi-number items separately, we classified the multi-number items into structural types, so, for example, items such as ‘one and two’ and ‘eight and six’ were both categorised as ‘number and number’. Then, we updated the Python script to capture the numbers in these multi-number items separately, and translated them into numerals. We did this until 20 or fewer cases of each structural type remained (an arbitrary threshold as it would have been impractical to account for every structural type due to the large size of the dataset). We excluded the remaining 2,078 multi-number items from the dataset. To finish, we constrained the numbers in our dataset to the range 0 to a billion, which led to the exclusion of 6,425 items.

This part of the procedure highlights a key limitation of an automatic approach to identifying numbers: when number words are written in nonstandard ways, or are not separated by commas, our automatic procedure may identify some of these numbers incorrectly (as blanket rules were established for how multi-number strings were dealt with, which may not be accurate for all cases), or these numbers may be excluded. Only a manual analysis would allow us to identify the numbers in these strings correctly in all instances. Ultimately, we believe that any numbers that are inaccurately identified will constitute noise in the data that should not affect the overall pattern of results we report, and that must be tolerated to facilitate the collection of such a large dataset.

Overall, out of 97,476,231 words in the BNC, the number identification procedure identified 1,739,343 numbers that met our search criteria. This figure constitutes 1.8% of the total number of words in the BNC mentioned above, but recall that some number word expressions comprised multiple words (e.g., ‘eight hundred’).

## 4 Results

### 4.1 Overall results

**Figure 1:**
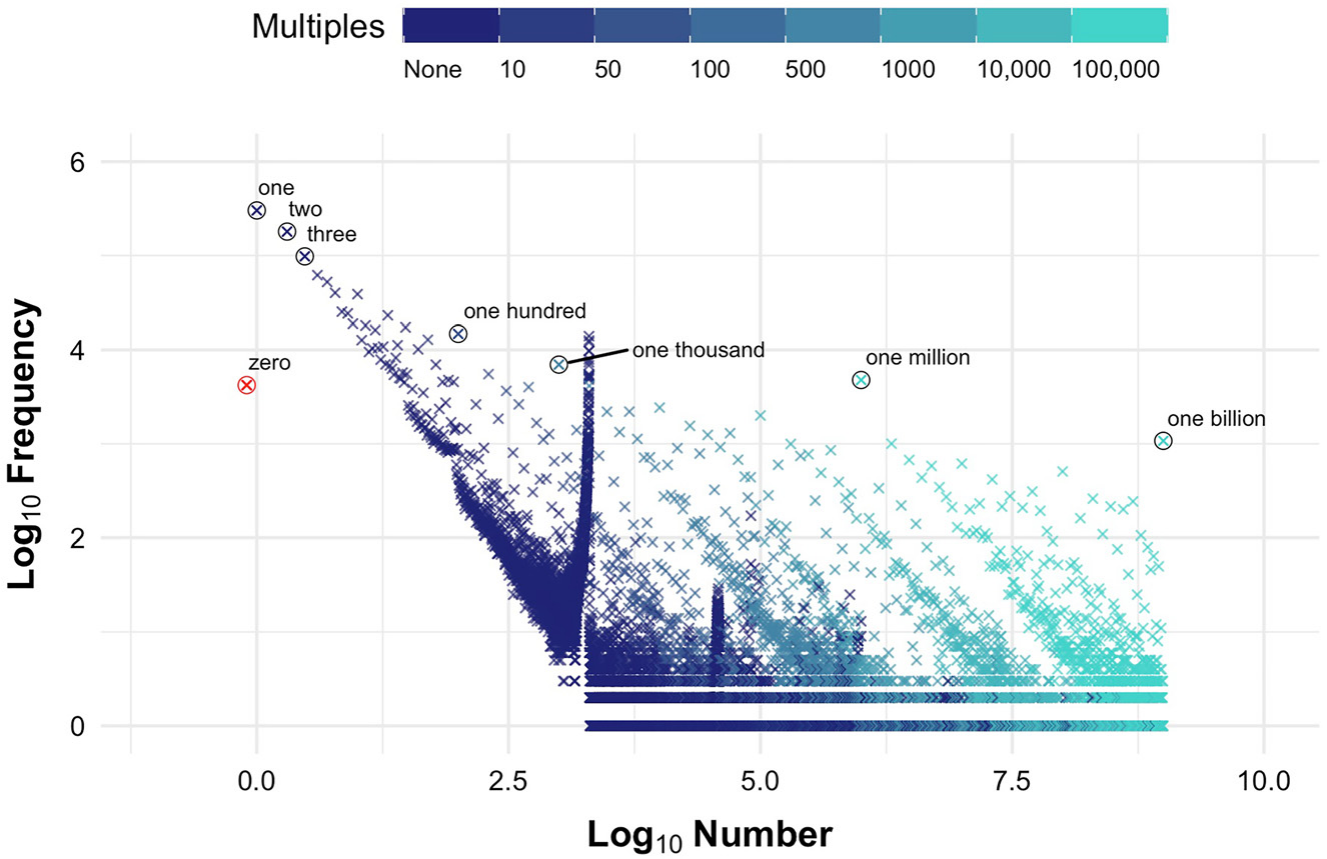
Frequencies for all integers that appear in the British National Corpus in any representational format. Both axes are base-10 logarithmically scaled (log_10_). Non-multiples and multiples of 10, 50, 100, 500, 1,000, 10,000, and 100,000 are color coded on a categorical scale from dark blue to light turquoise (see legend). The number 0 is in red to highlight that we have manually coded its log_10_ value as –0.1 to visualize it on a log_10_ scale, as the logarithm of 0 is not defined. The numbers 0, 1, 2, 3, 100, 1,000, 1,000,000, and 1,000,000,000 are circled and labelled.

First, we analysed the influence of magnitude, roundness, and cultural salience overall by looking at the number frequencies across the whole of the BNC. Figure 1 displays the frequencies for numbers from 0 to a billion, excluding decimals. Number and frequency are visualized on base-10 logarithmic scales (log_10_) – that is, *x* or *y* = 1 represents 10, *x* or *y* = 2 represents 100, *x* or *y* = 3 represents 1,000, *x* or *y* = 4 represents 10,000, and so on.

The downward trend shows that frequency decreases as numerical magnitude increases: smaller numbers are used more often than larger numbers. In fact, the number 1 is used more often than any other number, occurring 302,676 times (18.1% of all integer tokens). The second most frequent number is 2, which occurs 180,079 times (10.8% of all integer tokens), and the third most frequent number is 3, which occurs 98,035 times (5.9% of all integer tokens). As an exception to this trend, 0 is the 57th most frequent number in the dataset, occurring only 4,216 times (0.3% of all integer tokens). Zero is different from other integers because it usually represents the absence of a quantity, rather than the presence. Consequently, this number is not typically used in counting practices. It should, however, be noted that only the numeral ‘0’ and the number word ‘zero’ were considered in this study – words such as ‘none’ and ‘nought’ were not included.

The figure also shows that round numbers are used more often than unround numbers of a comparable magnitude. For example, focussing on the round numbers circled and labelled, 100 is used 14,819 times, while 99 is used 881 times and 101 is used 429 times; 1,000 is used 6,975 times, while 999 is used 182 times and 1,001 is used 54 times; 1 million is used 4,778 times, while 999,999 is used 13 times and 1,000,001 is used 9 times; and 1 billion is used 1,075 times, while 999,999,999 is not used at all.

The color of the data points denotes whether the numbers are non-multiples or multiples of round numbers of different magnitudes: 10, 50, 100, 500, 1,000, 10,000, and 100,000. The transition from dark blue to light turquoise rightward across the data points highlights the fact that, at higher magnitudes, the numbers that appear in the BNC are increasingly round numbers, and multiples of a larger round number (e.g., 110,000 is a multiple of 1,000 and 10,000, while 111,000 is a multiple of 1,000 but not 10,000). This result hints that people are more likely to round numbers at higher magnitudes, and to round to a greater extent – for instance, rounding 110,789 down to 110,000 (the nearest multiple of 10,000) instead of rounding it up to 111,000 (the nearest multiple of 1,000).

On top of the influence of magnitude and roundness, there are some other notable features of the figure. First, there is a spike in frequency starting at about *x* = 3 that reflects the high frequency of numerals from 1,000 to 2,000. Concordance lines extracted via the English Corpora interface (https://www.english-corpora.org/bnc/), which allows the user to conduct online searches of the BNC, showed that many of these numerals are years (e.g., ‘the year 1990’), and are used with increasing frequency as they approach the year the BNC’s collation was completed (1994). Following Pollman and Baayen (2001), we might infer that these numbers are used more frequently because recent dates are more relevant to discuss and so are more culturally salient. The second notable feature of the figure is the spike in frequency just before *x* = 5, which roughly represents the range 36,000–40,000. Concordance lines for the most frequent of these numbers (e.g., 38,166) show that many of these numbers are citations of page numbers mentioned frequently in multiple political and economic records from the Keesings Contemporary Archives in the informative writing subcorpus. The pages to which these numbers refer contain information about widely significant, relevant topics (e.g., the Kuwait Democratic Forum). The high frequency of these numbers appears to be an artifact of how the BNC’s compilers sampled texts to include in the corpus, rather than these numbers being culturally salient.

### 4.2 Statistical models

To understand the relative influence of magnitude, roundness, and cultural salience on number frequencies, we statistically modelled number frequency as a function of magnitude and roundness. We then identified the numbers used more frequently than predicted when controlling for magnitude and roundness, whose frequency may be attributable to cultural salience.

Bayesian negative binomial regression was used to model the number frequencies (see Winter and Bürkner 2021). We included numbers that do not appear in the BNC in this regression, which were assigned a frequency of 0, in addition to the numbers that were identified (i.e., ones that appear at least once). This step is justified because the frequency of the ‘missing’ numbers is genuinely 0 – it is not just that we did not identify them. Number frequencies are positive integers, and so are amenable to Poisson regression, which can be used to model unbounded count data. However, because there is more variance in these count data than what is expected under the Poisson distribution (known as overdispersion; see OSF repository for evidence of overdispersion in the data: https://osf.io/ze9vk/) negative binomial regression was used, which modelled both the mean and the variance in counts. Like Poisson regression, negative binomial regression uses the log link function (natural logarithm), which means that all coefficients reported below model the log_e_ number frequencies.

We modelled number frequencies for the range 1–1 million. We did not include 0 because logarithmically transforming 0 is not possible, and we imposed an upper limit of 1 million to mitigate the computational difficulties and time demands associated with analysing larger datasets in this manner (all numbers from 1 to 1 million were analysed, including numbers with a frequency of 0). We used default priors from the R package brms (Bürkner 2017, 2018) for the intercept and standard deviation. We set weakly informative priors on the independent variable slopes (normal distribution centered at 0, standard deviation of 0.5) to build “mild skepticism” (McElreath 2016, p. 186) into our analyses. Weakly informative priors bias slope estimates slightly towards zero, making our results more conservative when compared to a corresponding frequentist model.

The dependent variable in the model was Frequency and the independent variables were Log_10_ Number Magnitude and the roundness properties Multiple of 5, Multiple of 10, 10-ness, 2-ness, 2.5-ness, and 5-ness.3Technically, the mathematical definitions of 10-ness, 2-ness, 2.5-ness, and 5-ness proposed by Jansen and Pollmann (2001) mean that all numbers from 1 to 9 have 10-ness, because dividing these numbers by 1 (1 × 10^0^ = 1) equals an integer no greater than 9. Similarly, all multiples of 2 from 2 to 8 have 2-ness, because dividing these numbers by 2 (2 × 10^0^ = 2) equals an integer no greater than 9. As our treatment of roundness assumes that all round numbers are multiples of ten or five (although not all multiples of ten or five are necessarily round), we avoided this outcome by only considering the first power of ten and above for all roundness properties (rather than the zeroth). Variance Inflation Factors (VIFs) for the roundness properties in a linear regression model were all below 3, indicating that collinearity was not an issue (Winter 2019; see OSF repository for VIFs: https://osf.io/ze9vk/).4There is currently no implementation of VIFs in the brms R package, and in any case, whether the model is Bayesian or frequentist does not affect assessments of collinearity.


The coefficients from this model confirm that Log_e_ Frequency declines with Log_10_ Number Magnitude (*β* = –3.62, 95% *Bayesian credible interval* = [–3.65, –3.59]). From highest to lowest, the credible intervals for the roundness properties are: 10-ness (*β* = 4.46, 95% CI = [4.06, 4.88]), 2.5-ness (*β* = 3.84, 95% CI = [3.42, 4.29]), 5-ness (*β* = 3.39, 95% CI = [2.95, 3.87]), 2-ness (*β* = 2.74, 95% CI = [2.29, 3.20]), Multiple of 10 (*β* = 2.45, 95% CI = [2.38, 2.53]), and Multiple of 5 (*β* = 0.06, 95% CI = [–0.01, 0.13]). All the credible intervals are well above zero, except for Multiple of 5, which slightly overlaps with, but is mostly above, zero. All roundness properties thus predict that a number will be used more frequently, but Multiple of 5 much less so than the other roundness properties. Furthermore, numbers with more roundness properties (e.g., 200, which is a Multiple of 10, a Multiple of 5, and also has 10-ness, 2.5-ness, 5-ness, and 2-ness) are predicted to be used more frequently than numbers with fewer roundness properties (e.g., 15,000, which is a Multiple of 10, a Multiple of 5, and also has 2.5-ness and 5-ness). Moreover, some credible intervals do not overlap, indicating that some roundness properties are more predictive of number frequency than others, with 10-ness being the most predictive (more predictive than 5-ness, 2-ness, Multiple of 10, and Multiple of 5), followed by 2.5-ness (more predictive than 2-ness, Multiple of 10, and Multiple of 5), 5-ness (more predictive than Multiple of 10 and Multiple of 5), 2-ness and Multiple of 10 (both more predictive than Multiple of 5), and Multiple of 5.

We then identified numbers whose high frequency cannot be explained by either magnitude or roundness by examining the model residuals, which show how different the actual number frequencies are from the model’s predictions (see Winter 2019, Ch. 4). High residuals reflect numbers that are used more often than expected based on magnitude and roundness alone, factoring out the influence of the model’s predictors. To better understand why these numbers are used so frequently, we consulted concordance lines for them using the English Corpora interface (https://www.english-corpora.org/bnc/). A full list of the residuals is provided in the OSF repository for this paper (https://osf.io/ze9vk/).

The ten numbers with the largest residuals are all between 1984 and 1993, reflecting the prevalence of dates in the BNC, especially those recent to the completion of the BNC’s collation in 1994. If we exclude numbers between 1,000 and 2,000, the ten largest residuals are for numbers with at least one roundness property (1st: 250,000, 2nd: 300,000, 3rd: 12,000). If we exclude numbers with any roundness properties, the largest residual is for the number 80486, which refers to a microprocessor. The second largest residual is for the number 2001, which refers to the year and a savings plan called SaverPlus 2001. The third largest residual is for the number 999, which is one of the UK’s emergency services numbers. The numbers at ranks 8–10 in this list (38,211, 37,838, 37,914) are page numbers mentioned in texts from the Keesings Contemporary Archives.

### 4.3 Analysis of subcorpora

We now turn our attention to comparisons across and within the BNC subcorpora. To investigate the effect of register on number use, we compared more informational (presentational speech, e.g., lectures, educational demonstrations, classroom interactions; informative writing, e.g., non-fiction books about applied science, the arts, world affairs, etc.) and less informational contexts (conversational speech; imaginative writing, i.e., fiction books) within speech and writing respectively. Across these registers, we compared: (1) Numerical density – how often numbers are used in general; (2) Numerical diversity – how varied the set of numbers used is; (3) Decimals – how frequently decimals are used; and (4) Magnitude – how frequently smaller versus larger numbers are used. Then, in the written subcorpora, we investigated the format (numerals, number words, mixed numbers) used to represent numbers of different magnitudes.

#### 4.3.1 Numerical density

Relative to the size of each spoken subcorpus, people use numbers about the same amount in presentational speech (1.6%, 97,589 number tokens out of 5,987,379 words) and conversational speech (1.6%, 63,196 number tokens out of 3,976,158 words). Relative to the size of each written subcorpus, people use numbers more frequently in informative writing (2.1%, 1,473,425 number tokens out of 71,355,964 words) than in imaginative writing (0.7%, 105,133 number tokens out of 16,156,730 words). Thus, in writing, the more informational subcorpus (informational writing) is more numerically dense than the less informational subcorpus (imaginative writing), whereas in speech, there is not a substantial difference in numerical density between the more informational (presentational speech) and less informational (conversational speech) subcorpora.

#### 4.3.2 Numerical diversity

When people speak about numbers, they use more different numbers in presentational speech (1,633 types, type-token ratio [TTR] = 1.7%) than in conversational speech (634 types, TTR = 1.0%). When people write about numbers, they use more different numbers in informative writing (27,016 types, TTR = 1.8%) than in imaginative writing (1,412 types, TTR = 1.3%). Hence, in their respective modalities, the more informational subcorpora (presentational speech and informative writing) are more numerically diverse than the less informational subcorpora (conversational speech and imaginative writing).

#### 4.3.3 Decimals

When people talk about numbers, they use more decimals in presentational speech (1.0%, 978 decimals out of 97,589 number tokens) than in conversational speech (0.5%, 297 decimals out of 63,196 number tokens). When people write about numbers, they use more decimals in informative writing (4.6%, 68,257 decimals out of 1,473,425 numbers) than in imaginative writing (0.6%, 665 decimals out of 105,133 numbers). These results show that more numbers used in the more informational subcorpora (presentational speech and informative writing) are decimals than in the less informational subcorpora (conversational speech and imaginative writing) in their respective modalities.

#### 4.3.4 Magnitude

**Figure 2:**
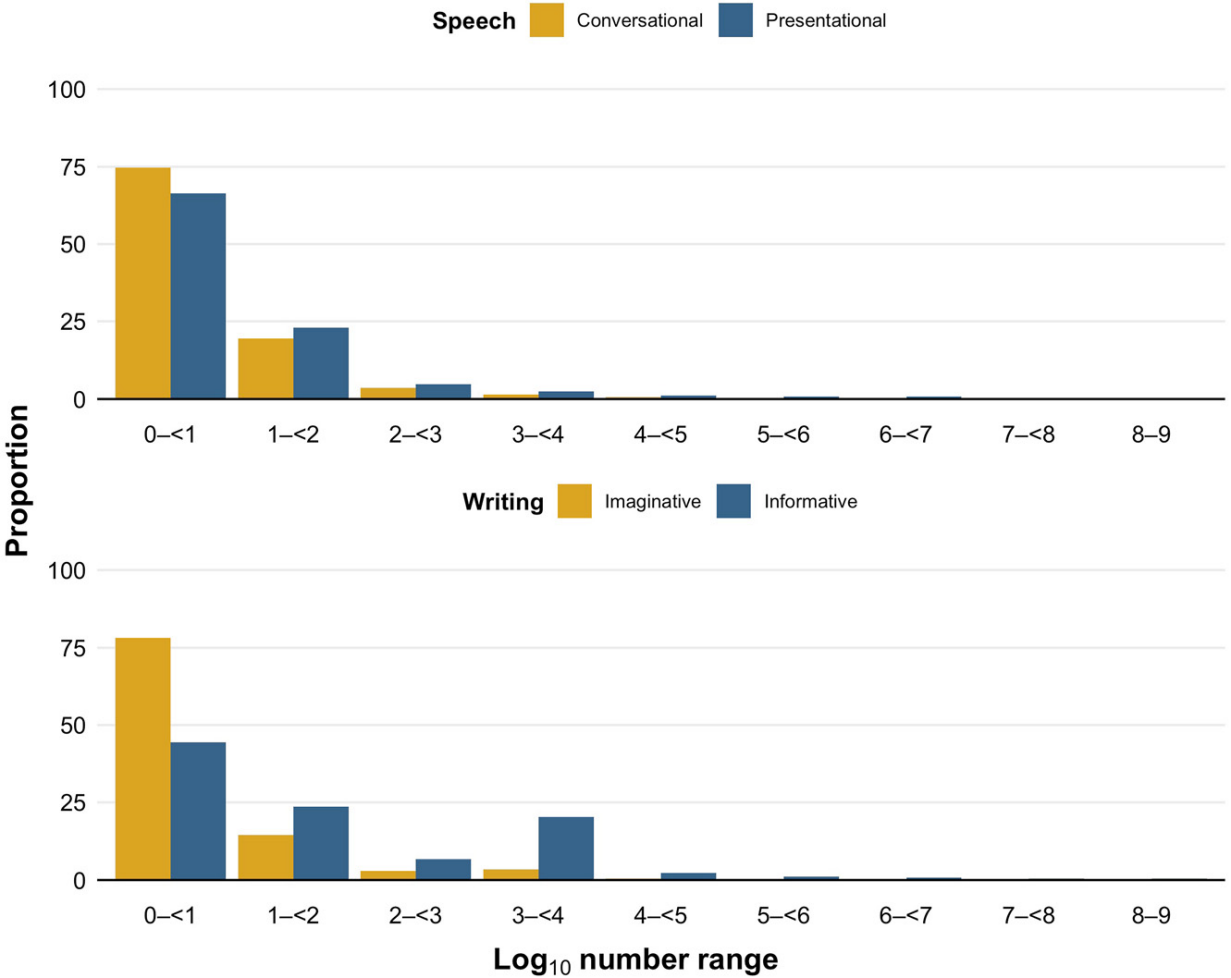
Proportions of number tokens in different log_10_ number ranges in the spoken and written subcorpora. Proportions are out of all number tokens in each subcorpus.

Figure 2 shows the proportion of number tokens in different log_10_ number ranges (i.e., magnitudes) in the different subcorpora. In all subcorpora, smaller numbers are used more often than larger numbers.

When we compare the subcorpora, we see that smaller numbers are proportionally more frequent in the less informational subcorpora compared to the more informational subcorpora in their respective modalities: the log_10_ number range 0–<1 (numbers 1–9) is proportionally more frequent for conversational speech (74.7%, 47,143 out of 63,091 tokens) than presentational speech (66.4%, 64,675 out of 97,332 tokens), and for imaginative writing (78.2%, 82,175 out of 105,045 tokens) than informative writing (44.4%, 649,597 out of 1,462,196 tokens).

In comparison, larger numbers are proportionally more frequent in the more informational subcorpora than in the less informational subcorpora in their respective modalities: the log_10_ number ranges 1–9 (numbers 10–1 billion) are proportionally more frequent for presentational speech (33.6%, 32,657 out of 97,332 tokens) than conversational speech (25.3%, 15,948 out of 63,091 tokens), and for informative writing (55.6%, 812,599 out of 1,462,196 tokens) than imaginative writing (21.8%, 22,870 out of 105,045 tokens).

The proportions of number tokens become increasingly small for the higher log_10_ number ranges as larger numbers are increasingly unlikely to be discussed in general, meaning that proportional differences between the subcorpora become trivially small. Also note that the relative increase for written numbers in log_10_ number range 3–<4 is due to numbers in the range 1,000–2,000 occurring frequently, often referring to years.

#### 4.3.5 Writing: representational formats

**Figure 3:**
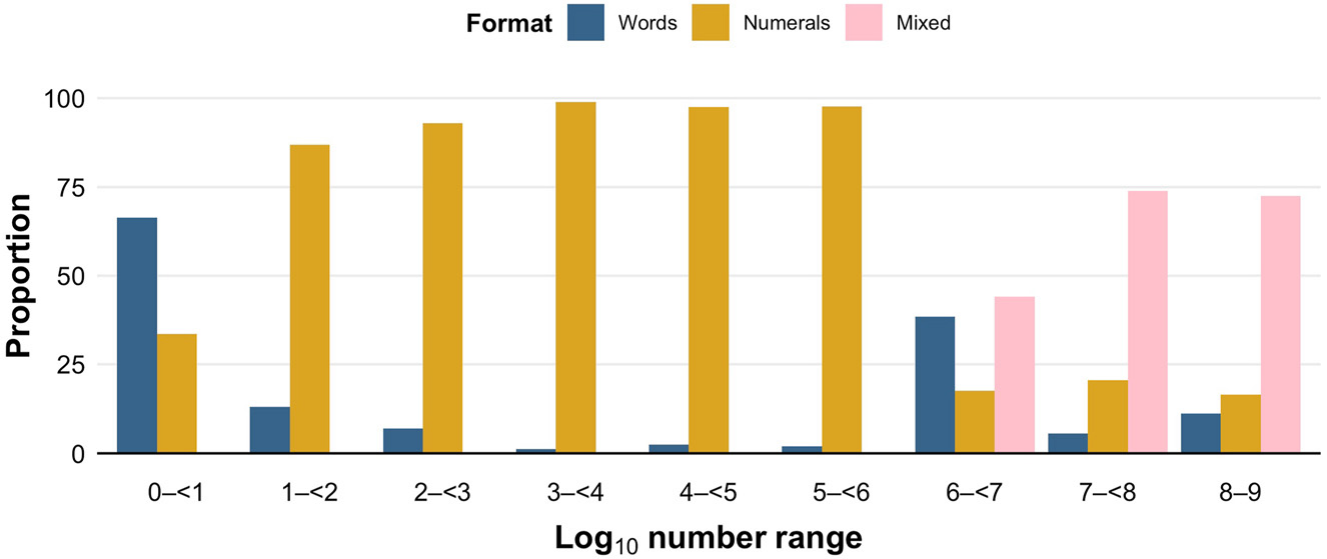
Proportions of number tokens in each log_10_ number range that were written in different representational formats. Proportions are out of all number tokens in each log_10_ number range.

Figure 3 displays the proportions of numbers in each log_10_ number range that were represented as a numeral, number word, or mixed number in writing. We focus on writing because the representational format of a number in writing is a decision made by the text’s author, whereas in the spoken texts, this format is a matter of transcription and so does not reflect how the number was spoken in context.

Overall, the figure shows a transition from the dominance of number words for the smallest numbers, to numerals for comparatively larger numbers, to mixed numbers for the largest numbers. In particular, numbers 1–9 (log_10_ number range 0–<1, 731,772 tokens) are mostly represented by number words (66.46%, 486,316 tokens) and less by numerals (33.54%, 245,456 tokens). Numbers 10–999,999 (log_10_ number range 1–<6, 811,536 tokens) are mostly denoted by numerals (92.77%, 752,834 tokens), followed by number words (7.23%, 58,647 tokens), and mixed numbers (0.01%, 55 tokens). Numbers 1 million–9,999,999 (log_10_ number range 6–<7, 11,888 tokens) are mostly represented by mixed numbers (44.03%, 5,234 tokens), followed by number words (38.45%, 4,571 tokens), and then numerals (17.52%, 2,083 tokens). Similarly, numbers 10 million–99,999,999 (log_10_ number range 7–<8, 6,413 tokens) are mostly denoted by mixed numbers (73.85%, 4,736 tokens), but, unlike the preceding number range, are denoted more by numerals (20.58%, 1,320 tokens) than by number words (5.57%, 357 tokens). This range is also more likely to be denoted by mixed numbers than the previous range. We see a similar pattern for numbers 100 million–1 billion (log_10_ number range 8–9, 5,632 tokens), which are mostly denoted by mixed numbers (72.46%, 4,081 tokens), followed by numerals (16.46%, 927 tokens), and then number words (11.08%, 624 tokens).

## 5 Discussion

This study is the largest, most comprehensive analysis of number use to date. Our results have a number of important implications. First, they confirm the findings of previous studies (Coupland 2011; Dehaene and Mehler 1992; Dorogovtsev et al. 2005; Jansen and Pollmann 2001) that frequency declines as numerical magnitude increases, and that round numbers are used more frequently than unround numbers of a similar magnitude. As discussed in detail in Section 2, small numbers may be used more often due to the ease of mentally processing these numbers (Dehaene and Mehler 1992), because they are more relevant to discuss (Cummins 2015: 32), and because we use scales that keep numbers small (Coupland 2011: 34–35). Round numbers may be used more frequently due to their psychological salience (Van der Henst and Sperber 2004) and cognitively accessibility or simplicity (e.g., Dehaene 1997), and also because the exact value may be unknown (Ruud et al. 2014) or irrelevant to discuss (e.g., Van der Henst et al. 2002). People may also use round numbers to avoid seeming pedantic (e.g., Lin 2013), and for strategic manipulation (Cummins and Franke 2021). However, our data do not allow us to differentiate between these different factors: we can state that smaller numbers and round numbers are used more often than larger and unround numbers, but our data do not tell us why.

Our findings clearly show that round numbers are not created equal: some are used more often and so may be seen as ‘rounder’ than others. First, even multiples of ten without 10-ness, 2-ness, 2.5-ness, or 5-ness (e.g., 810, 10,070) – and to a much lesser extent, multiples of five without 10-ness, 2-ness, 2.5-ness, or 5-ness (e.g., 815, 10,075) – are used more often than non-multiples^.^ Second, numbers with more roundness properties are more frequent than numbers with fewer roundness properties, extending Jansen and Pollman’s (2001) findings by showing that this result also applies if we treat being a multiple of ten or being a multiple of five as roundness properties. Third, supporting Cummins’ (2015: 34) speculation that some roundness properties may be more important than others in determining roundness, we find that 10-ness is the most important factor, followed by 2.5-ness, 5-ness, 2-ness and being a multiple of ten (which were tied), and, lastly, being a multiple of five.

**Figure 4:**
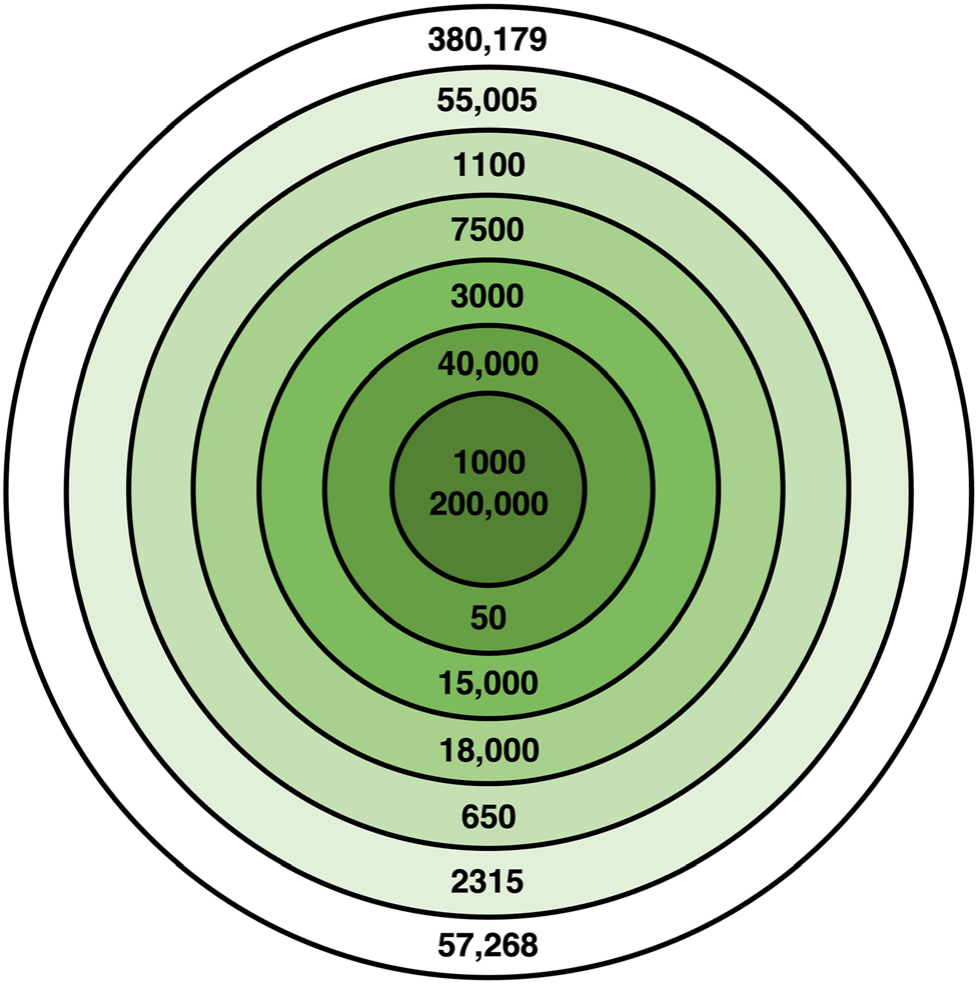
Roundness as a radial category, where green indicates roundness and white indicates the absence of roundness. The most prototypical or ‘roundest’ numbers are in the center circle (i.e., numbers with all six roundness properties: 10-ness, 2-ness, 2.5-ness, 5-ness, being a multiple of ten, and being a multiple of five). Progressing outward through the inner rings are less prototypical or ‘less round’ numbers with five, four, three, two, or one roundness properties. The outer ring shows numbers that have no roundness properties.

The concept of ‘roundness’ may thus not be either/or but rather may be a radial category with graded membership (Lakoff 1987, Ch. 6), consistent with prototype theory (Rosch 1973; Taylor 2003). Numbers with more roundness properties may be closer to the center of this category (i.e., be more prototypical of it) because they have more features associated with it (e.g., 10-ness, 2-ness), just as a robin is a more prototypical example of a bird than a penguin, because a robin has the feature ‘can fly’ that is associated with the *bird* category (e.g., Rosch 1975; Rossiter and Best 2013). A radial category of roundness is illustrated in Figure 4. A more complete depiction of roundness as a radial category would account for the different influence of different roundness properties (e.g., 10-ness > 5-ness, 2-ness, Multiple of 10, and Multiple of 5).

Our results also suggest that number use is less precise at higher magnitudes: people round larger numbers to a greater extent than smaller numbers (see also Coupland 2011). This aspect of numerical communication may reflect the less precise nature of numerical cognition at higher magnitudes (Cheyette and Piantadosi 2019; McCrink and Wynn 2007; Xu and Spelke 2000), which is captured by the Weber-Fechner law (DeWind et al. 2015; Shepard et al. 1975). Because this explanation appeals to the notion that we find quantities more difficult to discriminate between at higher magnitudes, it probably only applies to cardinal numbers (i.e., those denoting quantities) rather than, for example, the calendar years in our dataset, whose numerical value denotes their position in an ordinal sequence of years, rather than the number of years per se. For years, larger numbers (i.e., more recent years) may be easier to discriminate between than smaller numbers (i.e., less recent years), because more recent years may be easier to recall accurately. For example, while it may be easy to recall whether one went to Milan in either 2017 or 2018, it may be harder to recall whether one went to Rome in either 2007 or 2008.5We thank an anonymous reviewer for this point and example.


We also identified numbers that were used frequently because they are culturally salient. For example, confirming previous studies (Pollmann 1998; Pollmann and Baayen 2001), numbers between 1,000 and 2,000 were especially frequent as many were ordinal numbers that referred to years, particularly recent ones, which are usually more relevant to present discussion. The number 999 was also used relatively often in the BNC as it is one of the UK’s emergency services numbers, a nominal use of 999. Crucially, if the number 999 were not culturally salient, we would hardly expect it to be used at all, because in all other contexts it would probably be rounded to 1,000. Rather than reflecting cultural salience, other frequently used numbers seemed to reveal issues of unrepresentativeness in the BNC. The most obvious example is in the presence of ordinal page numbers from texts from the Keesings Contemporary Archives, which probably reflects the texts sampled for inclusion in the BNC rather than prominent use of these numbers outside this context. Other examples include the use of numbers as names for products, like the SaverPlus 2001 savings plan. While the nominal use of numbers in branding can certainly lead to numbers being culturally salient, such as 747 (Boeing jets), 501 (Levi jeans), and 57 (Heinz), it is doubtful whether the number 2001 is salient outside the BNC, at least not due to its use in this particular brand name. Future studies could conduct diachronic analyses to determine whether different numbers have gained or lost cultural salience over time. As cultures evolve, so too may the preference for communicating about different numbers, and the ways about which these numbers are communicated. For example, Chrisomalis (2020) demonstrates that the popularity of the expression ‘1.2 million’ has surpassed the equivalent expressions ‘twelve hundred thousand’ and ‘one million two hundred thousand’ over the years 1800–2000.

This study also investigated number frequencies across registers: across more informational (presentational speech, informative writing) and less informational (conversational speech, imaginative writing) contexts. We found that, in their respective modalities, the more informational subcorpora were more numerically diverse (people used more varied numbers) and contained more decimals and larger numbers. In writing, we found that informative writing was more numerically dense (people used more numbers in general) than imaginative writing. Overall, these measures suggest that numbers are integral to contexts in which communicating information is important. People may use numbers in informational registers to quantify phenomena for the benefit of one’s audience – such as when listing ingredients for a recipe (e.g., 2 tablespoons of tomato purée, ½ a teaspoon of dried marjoram) and describing how many people the meal will serve (e.g., 4 people) (see BBC Good Food 2022) – or to precisely record financial transactions (Coupland 2011). People may also use numbers to establish credibility (Koetsenruijter 2011) or convey an air of objectivity (Porter 1995). The fact that we found register differences is consistent with previous studies that found linguistic differences between English registers (Biber 2009a, 2009b; Biber et al. 1999; Biber and Clark 2002; Biber and Gray 2010; Conrad and Biber 2009; Egbert and Mahlberg 2020; Love et al. 2019; Neumann 2014).

Comparing representational formats in writing, we found that the numbers 1–9 were mostly represented as number words, whereas 10–999,999 were mostly represented as numerals, and 1 million–1 billion were mostly denoted by a mix of numerals and multiplier words, consistent with the recommendations of many writing style guides (e.g., Office for National Statistics 2022). These conventions may stem from the fact that larger numbers, especially unround numbers, are often longer to write and harder to parse when written as number words (e.g., ‘one hundred thousand, seven hundred and fifty five’) than as numerals (e.g., ‘100,755’). However, even numerals may be difficult to parse for very large numbers (e.g., ‘1,000,000,000’), which may explain why a mix of numerals and number words are preferred at these magnitudes (e.g., ‘1 billion’). Our results are consistent with Coupland’s (2011) in that we found that number words were used proportionally more often for smaller numbers. However, unlike this previous study, we found that number words were dominant over numerals for the range 1–9. This discrepancy may be attributable to the fact that the internet contains numerals from all languages, which was contrasted with number words in English, whereas our analysis compares numerals and number words used exclusively in English.

People talk and write about numbers in many different ways, which are difficult to account for exhaustively in a study like this that paints with broad brushstrokes to capture numerical communication on a large scale. In the interest of feasibility when working with large-scale data, blanket rules were established for what number expressions would be considered, which were necessarily imperfect. Our methodology automatically identified words that are typically used to denote numbers (e.g., ‘one’, ‘hundred’), but some numerical expressions were not identified (e.g., ‘nought’, ‘a dozen’, ‘a couple’), or were probably captured as separate numbers even though they were part of a larger expression (e.g., ‘two and a half thousand’ = ‘two’ and ‘thousand’; ‘one over three’ = ‘one’ and ‘three’). As a result, we did not examine more non-standard or colloquial ways of referring to numbers (e.g., ‘a couple hundred’), which may be more prevalent in speech than in writing, potentially leading us to underestimate number use in speech. Our dataset may also be slightly noisy or incomplete generally for the reasons described above. However, we believe that the results we report would hold with a more complete identification of numbers, due to the strength of the patterns observed and the sheer size of the dataset, and because our results replicate many of the patterns revealed in previous corpus analyses (Coupland 2011; Dehaene and Mehler 1992; Dorogovtsev et al. 2005; Jansen and Pollmann 2001).

To replicate our findings, it may be instructive to reproduce these analyses with another English language corpus, such as the Corpus of Contemporary American English (COCA; Davies 2008), or a corpus in a different language, for example, from the TenTen Corpus Family (Jakubíček et al. 2013). Doing so would reveal whether our results apply across language varieties – for example, from British (BNC) to American English (COCA) – and to other languages from the same (e.g., German) or a different (e.g., Arabic) language family. It may also reveal differences in the numbers that are salient across cultures. For example, we might expect 911 (rather than 999) to be frequent in COCA, as 911 is the US’s emergency services number. Other corpora also have a different makeup of registers than the BNC, affording different kinds of comparative analyses than the more versus less informational comparison reported here. A replication could also be conducted with the 2004 version of the BNC (Brezina et al. 2021; Love et al. 2017) when the full written English texts are downloadable (only the spoken English texts are currently available).

People use some numbers more often than others. This large-scale study has shed light on the numbers people use more often, in what contexts, and why, in connection to magnitude, roundness, cultural salience, and register, and has identified the formats with which writers choose to represent these numbers. In doing so, it has constructed a detailed profile of number use in spoken and written English.
